# Depleting interferon regulatory factor‐1(IRF‐1) with CRISPR/Cas9 attenuates inducible oxidative metabolism without affecting RA‐induced differentiation in HL‐60 human AML cells

**DOI:** 10.1096/fba.2020-00004

**Published:** 2020-05-22

**Authors:** Kaiyuan Zhu, Jianbo Yue, Andrew Yen

**Affiliations:** ^1^ Department of Biomedical Sciences Cornell University Ithaca NY USA; ^2^ City University of Hong Kong ShenZhen Research Institute ShenZhen China; ^3^ Department of Biomedical Sciences City University of Hong Kong Hong Kong China

**Keywords:** differentiation, HL‐60 cells, inducible oxidative metabolism, interferon regulatory factor‐1(IRF‐1), retinoic acid

## Abstract

The known collaboration between all‐transretinoic acid and interferon motivates this study of the dependence of RA‐induced leukemic cell differentiation on interferon regulatory factor‐1 (IRF‐1), a transcription factor that is the main mediator of interferon effects. In the HL‐60 acute myeloid leukemia (AML) model that represents a rare RA‐responsive subtype of AML, IRF‐1 is not expressed until RA induces its prominent expression, and ectopic IRF‐1 expression enhances RA‐induced differentiation, motivating interest in how IRF‐1 is putatively needed for RA response. Accordingly, we created CRISPR/Cas9‐mediated IRF‐1 knockout HL‐60 cells. Contrary to expectation, loss of IRF‐1 did not diminish RA‐induced cellular signaling that propels differentiation, and RA‐induced cell differentiation markers, including CD38 and CD11b expression and G1/G0cell cycle arrest, were unaffected. However, elimination of IRF‐1 inhibited RA‐induced p47phox expression and inducible oxidative metabolism detected by reactive oxygen species (ROS), suggesting IRF‐1 is essential for mature granulocytic inducible oxidative metabolism. In the case of 1,25‐Dihydroxyvitamin D3‐induced differentiation to monocytes, IRF‐1 loss did not affect D3‐induced expression of CD38, CD11b, and CD14, and G1/0 arrest; but inhibited ROS production. Our data suggest that IRF‐1 is inessential for differentiation but upregulates p47phox expression for mature‐cell ROS production.

**Abbreviations**
1,25(OH)2D3: 1,25‐Dihydroxyvitamin D3
AML: acute myeloid leukemia
APL: acute promyelocytic leukemia
DSB: double‐strand break
FAB: French American British scheme
ICSBP: IFN consensus‐binding protein
IFN‐β: interferon‐β
IRF‐1: interferon regulatory factor‐1
KO: knockout
MAPK: mitogen‐activated protein kinase
NBT: nitroblue tetrazolium
NC: negative control
NLS: nuclear localization sequence
PML‐RARα: promyelocytic leukemia‐retinoic acid receptor alpha
RA: retinoic acid
RAR/RXR: retinoic acid receptor/retinoid X receptor
ROS: reactive oxygen species
SEM: standard error of the mean
SFKs: Src family kinases
TPA: 12‐o‐tetradecanoylphorbol‐13‐acetate
WT: wild type

## 1 INTRODUCTION

Retinoic acid (RA), a derivative of vitamin A, is a fundamental biological regulator of cell proliferation and differentiation in embryogenesis that became a prominent therapy for acute promyelocytic leukemia (APL), which is a t(15,17)‐positive M3 subtype of acute myeloid leukemia (AML) in the French American British scheme (FAB M3).^1^, ^2^ Once a fatal disease, APL, is considered as the most curable subtype of adult leukemia, with a 90% remission rate. This is in contrast to most AML which have very poor remission and survival rates. APL responds to RA, a noncytotoxic chemotherapy drug that induces differentiation and cell cycle arrest. RA‐based therapy used as a differentiation therapy, with arsenic trioxide to maintain the remission, is the current standard of care for the treatment of APL patients.^3^ RA induces the differentiation of leukemic promyelocytes into mature granulocytes.^4^ Besides RA, 1,25‐Dihydroxyvitamin D3(1,25(OH)2D3) is also able to initiate the differentiation of promyelocytic leukemia cells but to monocyte‐like cells instead of granulocytes. Nevertheless, D3 is not clinically used because of hypercalcemic toxicity.^5^, ^6^ However, RA monotherapy is dogged by the occurrence of relapse where recurrence of disease is now associated with resistance to RA. RA treatment also can induce a cardiopulmonary sequela, RA syndrome, that can be fatal. Finally, although APL responds to RA, most AML do not.^7^, ^8^ Hence, there is great interest in combination therapy that exploits molecular mechanistic insights into the mechanism of action of RA to devise combination therapies that might reduce the RA dosage and overcome resistance, as well as extend efficacy beyond APL to other AMLs.

Interferon‐γ and RA are known to cooperate in regulating cellular effects, including immune response, in hematopoietic and other cells.^9^ The historically dominant paradigm is that the IRF‐1 transcription factor, the main effector of interferon action, has nuclear functions that support RA‐activated retinoic acid receptor/retinoid X receptor (RAR/RXR)‐induced transcriptional activation needed for myelopoiesis.^10^ IRF‐1 was first identified in 1988. It mediated the transcription of interferon‐β (IFN‐β) upon virus infection.^11^ IRF‐1 is composed of a well‐conserved N terminal DNA binding domain and C‐terminal activation domain governed by phosphorylation. The 325 aa human protein has two nuclear localization sequence (NLS) motifs (amino acid 120‐140) that regulate its nuclear translocation.^12^, ^13^ IRF‐1 acts as a tumor suppressor in various types of cancer, especially in human leukemia. The IRF‐1 gene is located in the chromosome 5q31 region, where its deletion is a prominent feature of many leukemias.^14^ Besides antitumor activity, IRF‐1 also regulates hematopoiesis.^15^ It is ergo an attractive candidate to probe as an effector/enhancer of RA antileukemic action.

Cell line models have been potent tools for experimentally querying the significance of specific genes in a putative signaling pathway because of their susceptibility to experimental manipulation of gene expression. HL‐60 cells are a patient‐derived lineage bipotent GM‐precursor cell recently found to bear fidelity to an RA‐responsive, t(15,17)‐negative subtype of AML.^16^ RA induces their myeloid differentiation, whereas 1,25‐dihydroxyvitamin D3(1,25(OH)_2_D3) induces their monocytic differentiation.^17^ Historically the presence of the t(15,17) translocation that generates the promyelocytic leukemia‐retinoic acid receptor alpha (PML‐RARα) fusion protein is the sine qua non for RA response in AML, hence these cells engender particular interest for molecular mechanistic analysis of RA response in AML.^18^ In particular, they are of interest for deriving molecular mechanistic insights to circumvent RA resistance in AML. One such insight is that RA‐induced differentiation/arrest, and specifically RA‐induced transcriptional activation by RAR/RXR, is enabled by a MAPK pathway‐related signal that originates from a macromolecular signalsome in the cytoplasm that forms in response to RA and discharges components to the nucleus resulting in nuclear enrichment of canonically cytosolic signaling molecules, including Raf‐1. Previous reports have identified components of this signalsome, such as the CD38 membrane receptor, MAPK signaling axes Raf/Mek/Erk, Src family kinases, the Vav GEF, c‐CBL, 14‐3‐3, and SLP‐76 adaptors, and interestingly the IRF‐1 transcription factor.^19^, ^20^, ^21^, ^22^, ^23^, ^24^, ^25^, ^26^, ^27^ However, the trigger that initiates formation of the signalsome that generates the signal driving differentiation remains elusive. Interferon regulatory factor 1 (IRF‐1), a transcription factor that is induced by RA in HL‐60 AML, as well as NB4 APL cells, is a candidate.^28^, ^29^ In untreated cells it is not expressed. RA induces prominent expression. Ectopic expression of IRF‐1 results in enhanced RA‐induced differentiation of the stable transfectants compared to parental wild‐type cells.^23^ Hence, IRF‐1 is a known collaborator with RA, a component of the signalsome that propels RA‐induced differentiation, and its increased expression enhances RA‐induced differentiation. This makes IRF‐1 a molecule of interest to study for its role in RA‐induced leukemia cell differentiation. In this study, we depleted IRF‐1 in HL‐60 cells using the CRISPR‐Cas9 system. Unexpectedly, we found that signaling thought to propel differentiation and the cell surface differentiation markers induced either by RA or 1,25(OH)_2_D3 were not affected in these IRF‐1 KO HL‐60 cells; but RA‐induced inducible oxidative metabolism, a functional differentiation marker for fully mature myelo‐monocytic cells, was crippled by loss of IRF‐1. While IRF‐1 is surprisingly not a trigger for signaling and ensuing differentiation, it may be necessary for the last late stages of differentiation or just the feature of induced superoxide production.

## 2 MATERIALS AND METHODS

### 2.1 Cell culture

HL‐60 human myeloblastic leukemia cells derived from the original patient isolates was a generous gift of Dr Robert Gallagher, certified and tested for mycotoxin by Bio‐Synthesis, Lewisville, TX, USA, in August 2017. Wide‐type, CRISPR‐mediated HL‐60 cells were cultured in RPMI 1640 supplemented with 5% heat‐inactivated fetal bovine serum (GE Healthcare) and 1% antibiotic/antimycotic (Thermo Fisher Scientific) in a 5% CO_2_ humidified atmosphere at 37°C. HL‐60 cell cultures were passed every 2 or 3 days to avoid cell densities over 1 × 10^6^ mL. Retinoic acid was added from a stock of 5 mmol/L in ethanol to make a final concentration of 1μ mol/L and 1,25‐dihydroxy vitamin D3 was added from a stock of 1 mmol/L in ethanol to make a final concentration of 0.5 μmol/L.

### 2.2 Construction of CRISPR‐mediated stable cell lines

Three pairs of sgRNA‐targeting IRF‐1 and one pair of negative control sgRNA were cloned into pLenti‐CRISPRv2 plasmid (Addgene #52961; Addgene) following the depositor's protocol. The primer sequences for the CRISPR plasmids are as follows: KO1 (Forward: 5'‐CACCGCTCATGCGCATCCGAGTGAT‐3', Reverse: 5'‐AAACATCACTCGGATGCGCATGAGC‐3'); KO2 (Forward: 5'‐CACCGCTCCCTGCCAGATATCGAGG‐3', Reverse: 5'‐AAACCCTCGATATCTGGCAGGGAGC‐3'); KO3 (Forward: 5'‐CACCGTTAATTCCAACCAAATCCCG‐3', Reverse:5'‐AAACCGGGATTTGGTTGGAATTAAC‐3'); and Negative Control (Forward: 5'‐CACCGGTTCCGCGTTACATAACTTA‐3', Reverse: 5'‐AAACTAAGTTATGTAACGCGGAACC‐3').

The viral transduction, selection, and production of stable transfectants from pooled cells were as described before. Pooled cells after viral transduction and selection were used for experiments in order to avoid the possibility of clonal bias.^30^

### 2.3 Genomic cleavage detection assay

The assays were conducted with the GeneArt Genomic Cleavage Detection Kit (Thermo Fisher Scientific) following the manufacturer's protocol. The genomic DNA was extracted from wide‐type and the CRISPR‐mediated cells. Primers were designed to amplify approximately 500bp fragments covering the genomic loci where the IRF‐1 sgRNA targeted. The primer sequences are as follows: primer 1 (Forward: 5'‐ATCCTGAAGCCATCACTTGC‐3', Reverse: 5'‐CTTCCCTTTTTGAGCTGCAT‐3'); primer 2 (Forward: 5'‐TTGACCACTGTGGCTCTCTG‐3', Reverse: 5'‐TGGCCTTGCTCTTAGCATCT‐3'). Primer 1 was used to amplify the genomic DNA fragments from KO1 and KO3 cells, while primer 2 was used to amplify the genomic DNA fragment from KO2 cell.

### 2.4 Western blot analysis and antibodies

Cells were harvested by centrifugation at 120 *g* for 5 minutes in a microfuge. The pellets were washed with PBS and lysed with mammalian protein extraction reagent (Pierce) with protease and phosphatase inhibitors (Sigma). The lysates were frozen at −80°C overnight and defrosted on ice, then cleared by centrifugation at 16 060 *g* for 10 minutes at 4°C. The supernatants were collected, and protein concentrations were determined with the Pierce BCA Protein Assay (Thermo Fisher Scientific). Equal amounts of total protein lysates (30 μg) were resolved by SDS‐PAGE, electrotransferred onto PVDF membranes, probed with antibodies, and detected with ECL reagent (GE Healthcare). The antibodies used for western blots are as follows: IRF‐1 was from BD Biosciences (San Jose, CA); GAPDH, c‐Raf, Lyn, Fgr, p47phox, Slp‐76, Vav1, PU.1, horseradish peroxidase anti‐mouse, and anti‐rabbit antibodies were from Cell Signaling (Danvers, MA); and c‐Cbl was from Santa Cruz Biotechnology (Santa Cruz, CA). The relative intensity of specific band was calculated against GAPDH using ImageJ software.

### 2.5 Flow cytometric phenotypic analysis

For the phenotypic analysis, cultures of HL‐60 cells were initiated at a density of 0.1 × 10^6^ mL on day 0. The cells were treated with either 1 μmol/L RA (Sigma) or 0.5 μmol/L 1,25‐dihydroxyvitamin D3 (Cayman) to induce differentiation. For the staining of CD38, CD11b, and CD14, 1 × 10^6^ cells were collected and centrifuged at 120 × *g* for 5 minutes. The cell pellets were resuspended in 200 μL PBS with 2.5 μL of APC‐conjugated CD11b antibody, PE‐conjugated CD38 antibody or PE‐conjugated CD14 antibody (all from BD Biosciences) at 37°C for 1 hour and analyzed with an LSR II flow cytometer (Becton Dickinson). For the cell cycle analysis, the same number of cells was centrifuged at 120 *g* for 5 min, and stained by resuspension in PI solution (50 mg/mL propidium iodine, 1 ml/mL Triton X‐100, and 1 mg/mL sodium citrate), stored at 4°C overnight, and then analyzed by flow cytometry. Gating was set to exclude debris and doublets.

### 2.6 Measurement of inducible ROS

Cytoplasmic Superoxide was detected by its capability to reduce soluble NBT to a blue‐black precipitate, formazan as reported before.^31^ 1 × 10^6^ cells were collected and centrifuged at 120 × *g* for 5 minutes. The cells were resuspended with 0.2 mL of 12‐o‐tetradecanoylphorbol‐13‐acetate (TPA)‐nitroblue tetrazolium (NBT) stock and incubated at 37°C for 20 minutes. The fraction of cells containing the cytoplasmic, blue‐black precipitate, and formazan was scored using a hemacytometer. The working concentration of NBT (Sigma) is 2 mg/mL, which was diluted in PBS and protected from light. The working concentration of TPA (Sigma) is 0.2 μg/mL, which was diluted in DMSO.

### 2.7 Statistical analysis

*P*‐values between treatment group means were calculated using ANOVA within GraphPad software. The data represent the means of three repeats ± SE of the mean (SEM). A *P* < .05 was considered significant.

## 3 RESULTS

### 3.1 Depletion of IRF‐1 with the CRISPR/Cas9 system in HL‐60 cells

**FIGURE 1:**
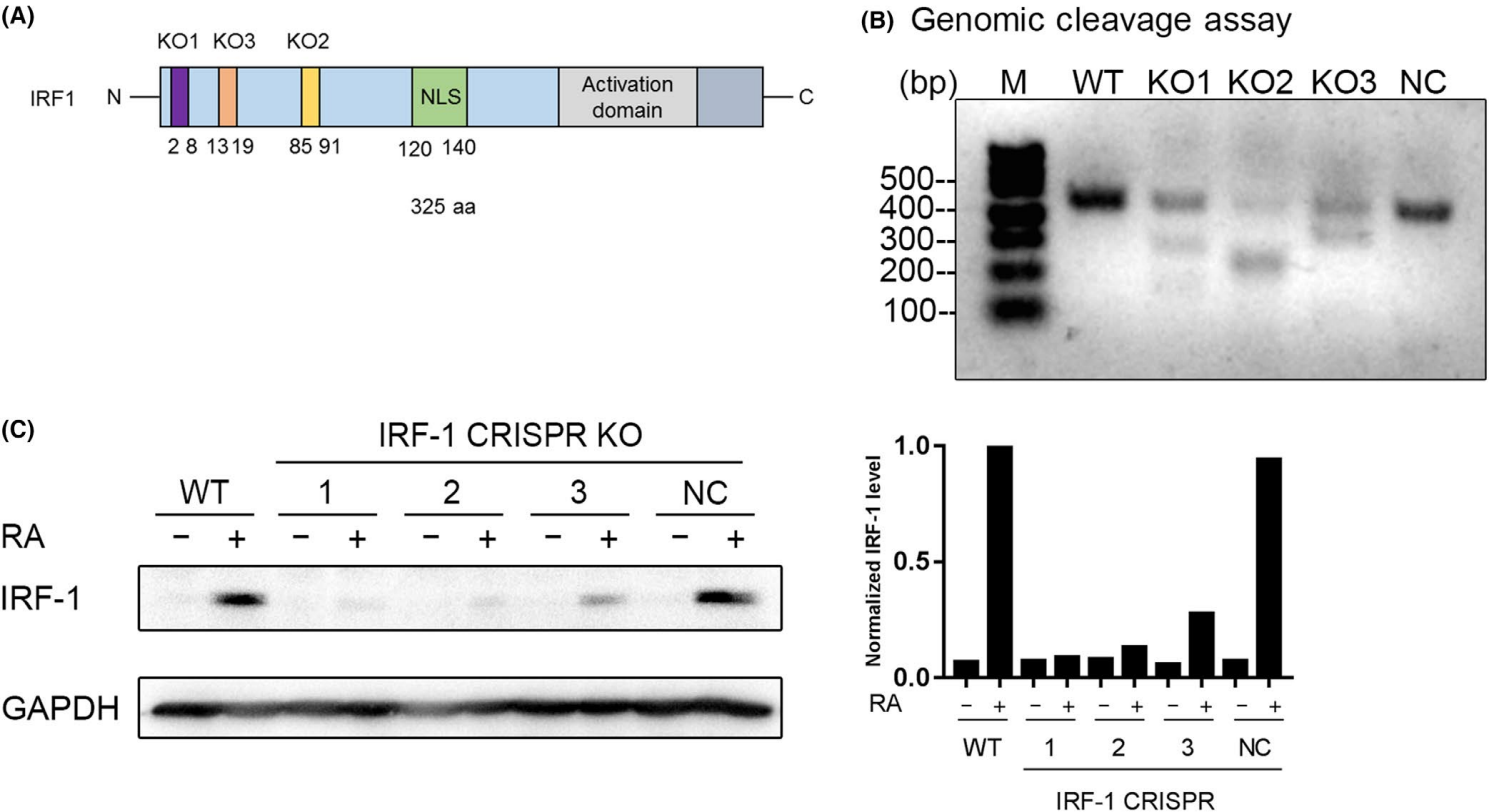
Depletion of interferon regulatory factor‐1 (IRF‐1) with the CRISPR/Cas9 system in HL‐60 cells. A, A schematic of IRF‐1 structure, showing where the three pairs of sgRNA target the exons of IRF‐1 before the NLS. B, Genomic cleavage detection assay that analyzed the cleavage sites of the three IRF‐1 sgRNAs. (C) Western blot of IRF‐1. Wild‐type and CRISPR‐derived HL60 cells were treated with 1 μmol/L RA as indicated for 48 h and the cell lysate was collected for western blot analysis. The relative level of IRF‐1 against GAPDH was calculated with ImageJ

To knockout IRF‐1 in HL‐60 cells, we applied CRISPR/Cas9‐mediated gene editing. To abrogate IRF‐1’s ability to regulate gene transcription, three sets of sgRNA (KO1, KO2, and KO3) were designed to cleave the exons of IRF‐1 before its NLS sequence. A pair of nontargeting sgRNA was included as the negative control (NC; Figure 1A). To confirm the CRISPR‐Cas9 gRNA‐cleaved targeted specific sites, we used Genomic Cleavage Detection Kit to detect the genome cleavage. Primers initiating from the introns and ending at the exons of IRF‐1 were designed and yielded a specific band around 500bp from the genomic DNA of wild‐type (WT), IRF‐1 KO1, KO2, KO3, and NC HL‐60 cells (Figure S1). The fragments were then used for the cleavage detection assay. We found that unlike WT and NC cells, the genomes of KO1, KO2, and KO3 cells were specifically cleaved by the IRF‐1 CRISPR‐Cas9 gRNAs (Figure 1B). Finally, we measured the levels of IRF‐1 protein in the CRISPR‐Cas9 knockout cells. As reported before, the expression of IRF‐1 was silent in untreated HL‐60 cells, and it was induced by RA treatment.^23^ We found that 1 μmol/L RA treatment for 48 hours dramatically enhanced the protein level of IRF‐1 in WT and NC cells. But the RA‐induced expression of IRF‐1 was grossly inhibited in KO1, KO2, and KO3 cells (Figure 1C). Taken together, we conclude that we created HL‐60 sublines where the CRISPR‐Cas9 KO successfully essentially eliminated IRF‐1 expression.

### 3.2 Depletion of IRF‐1 exerted little effect on RA‐induced signalsome

**FIGURE 2:**
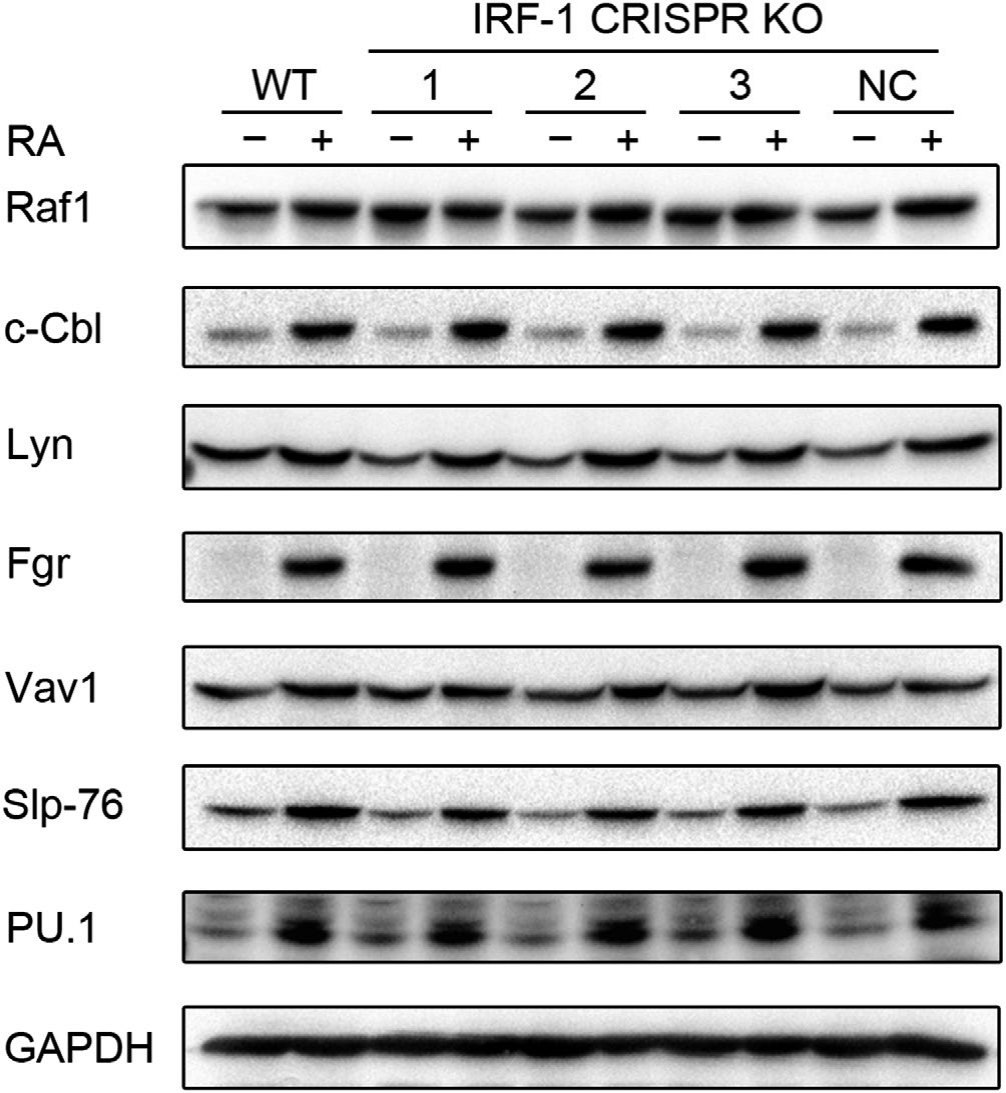
Depletion of interferon regulatory factor‐1 (IRF‐1) exerted little effect on RA‐induced changes in signalsome components or on PU.1. Wild‐type and CRISPR‐derived HL‐60 cells were treated with 1 μmol/L RA as indicated for 48 h, the cells were collected for western blot analysis of RA‐induced signalsome components and PU.1

Using the IRF‐1 KO cells, we then determined the effect of losing IRF‐1 on RA‐induced cellular signaling. Unexpectedly, we found that depletion of IRF‐1 had little effect on RA‐induced upregulation of an ensemble of signalsome components which have been reported to play essential roles in RA‐induced myeloid differentiation. In particular, expression of Raf‐1 was unaffected. RA‐induced upregulation of Src Family kinases (SFKs), Fgr and Lyn, was also unaffected by loss of IRF‐1, as was expression of the GEF, Vav, and the adaptor Slp‐76. These are signalsome components that have been implicated as drivers of myeloid differentiation. For instance, Raf‐1, which activates the Raf/Mek/Erk axis of MAPK signaling, promoted RA‐induced cell differentiation through translocation into the nucleus to enable transcriptional activation by RAR/RXR. Ectopic expression upregulating Raf‐1 enhanced RA‐induced signaling and differentiation, consistent with a role for it in driving differentiation.^32^, ^33^ Raf‐1 exists with c‐Cbl in the signalsome. Furthermore, in another cell Raf‐1 and c‐Cbl also both bind 14‐3‐3, another signalsome component, consistent with their interaction.^32^, ^33^, ^34^, ^35^ C‐Cbl, an adaptor and E3‐ligase, was also found to enhance RA‐induced differentiation when expression was enhanced by ectopic expression. C‐Cbl is an adaptor connected to CD38, and CD38 expression drove signaling and differentiation.^24^ Lyn and Fgr were upregulated by RA treatment and pharmacologically enhancing their expression/activation was associated with enhanced differentiation.^35^ They also played roles in protecting cells from apoptosis after RA treatment.^36^ SLP‐76 expression also drove RA‐induced differentiation.^22^, ^26^ These molecules interacted in the signalsome as demonstrated by immunoprecipitation and FRET; and IRF‐1 immunoprecipitated with c‐Cbl consistent with IRF‐1 intimacy with the workings of the signalsome. Moreover, IRF‐1 has been reported to mediate granulocyte differentiation through inducing PU.1 transcription. IRF‐1 null mutation led to PU.1 silencing and impaired granulocytic maturation in mice.^37^ However, we found that the depletion of IRF‐1 did not affect RA‐induced PU.1 expression in HL‐60 cells (Figure 2; Figure S2). Taken together, disruption of the IRF‐1 transcription factor surprisingly did not affect major signalsome components putatively propelling differentiation after RA treatment in HL‐60 cells.

### 3.3 Depletion of IRF‐1 exerted little effect on RA‐induced differentiation

**FIGURE 3:**
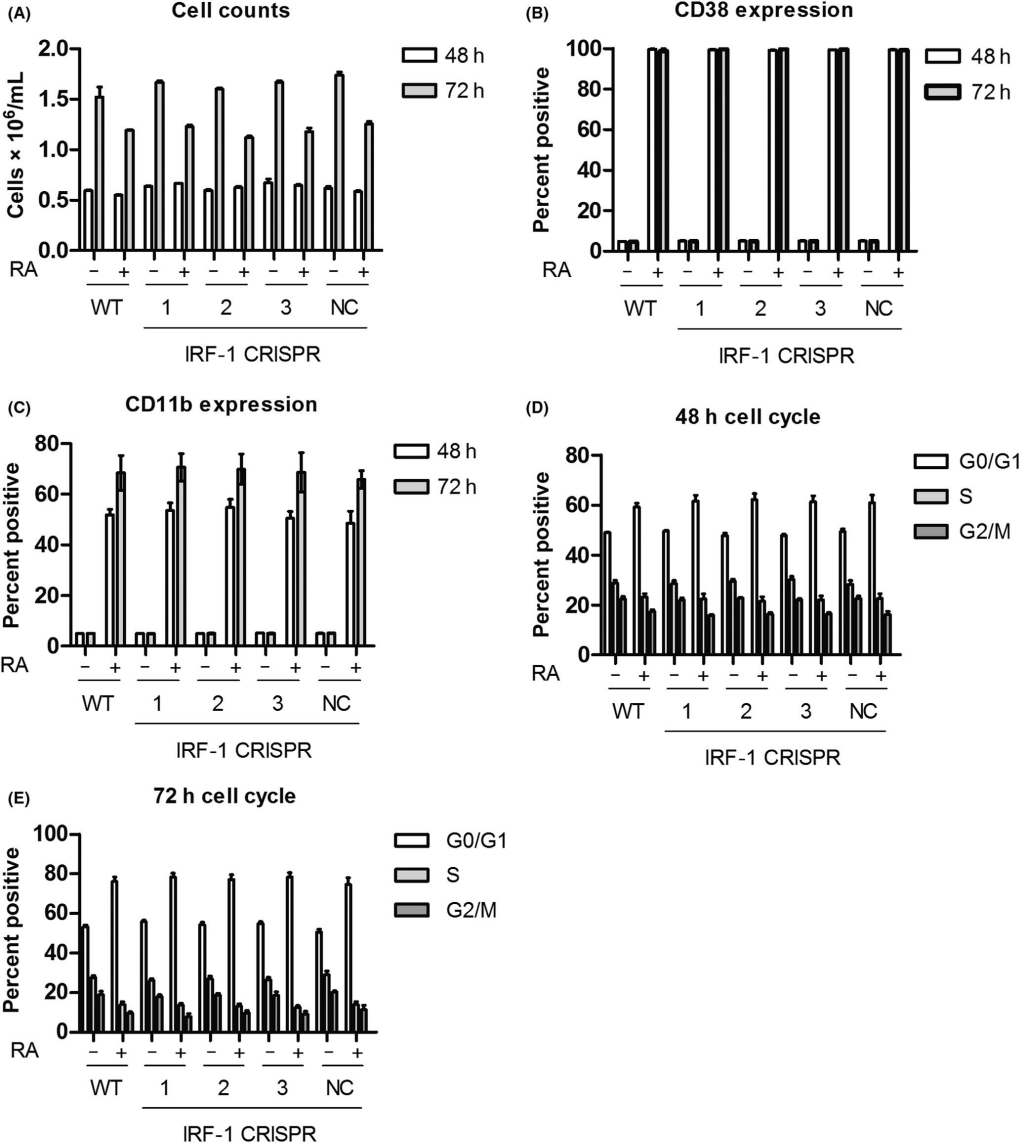
Depletion of interferon regulatory factor‐1 (IRF‐1) had little effect on RA‐induced differentiation. Wild‐type and CRISPR‐derived HL‐60 cells were treated with 1 μmol/L RA as indicated for 48 and 72 h, the cells were collected for analysis of (A) cell number, (B) CD38 expression, (C) CD11b expression, (D) 48 h cell cycle, and (E) 72 h cell cycle phase distribution

To investigate whether depletion of IRF‐1 affected RA‐induced cell differentiation, we measured the common markers for myeloid differentiation. The cell density, CD38 expression, CD11b expression, and G1/G0 cell cycle arrest at 48 and 72 hours following 1 μmol/L RA treatment were determined. We found that the IRF‐1 CRISPR knockout cells had a similar pattern of induced differentiation by these indexes when compared with the WT or NC HL‐60 cells after RA treatment (Figure 3). We explored the possibility that 0.1 μmol/L RA would provide a weaker differentiation driving stimulus than 1 μmol/L RA and might reveal the influence of losing IRF‐1 on differentiation which the higher dose might not be sensitive to. For 0.1 μmol/L RA‐treated WT cells, CD38 was still 100% after 48 and 72 hours of treatment—as it was for 1 μmol/L. The percentage of CD11b‐positive cells was decreased from around 50% (1 μmol/L) to 25% (0.1 μmol/L) at 48 hours; and 70% (1 μmol/L) to 40% (0.1 μmol/L) at 72 hours following RA treatment. Similarly, G1/G0 cell cycle arrest was also attenuated. RA‐induced G1/G0 accumulation dropped from 60% (1 μmol/L) to 50% (0.1 μmol/L) at 48 hours; and 80% (1 μmol/L) to 60% (0.1 μmol/L) at 72 hours following RA treatment (Figure S3). Hence, the lower dose elicited a weaker response, indicating that the RA dose was limiting and not saturating cellular response. However, the response of the IRF‐1 knockout cells at this RA dose was indistinguishable from the parental WT cells. Taken together, these data suggest that IRF‐1 is not required for RA‐induced HL‐60 cell differentiation.

### 3.4 Depletion of IRF‐1 suppressed RA‐induced ROS through decreasing p47phox expression

**FIGURE 4:**
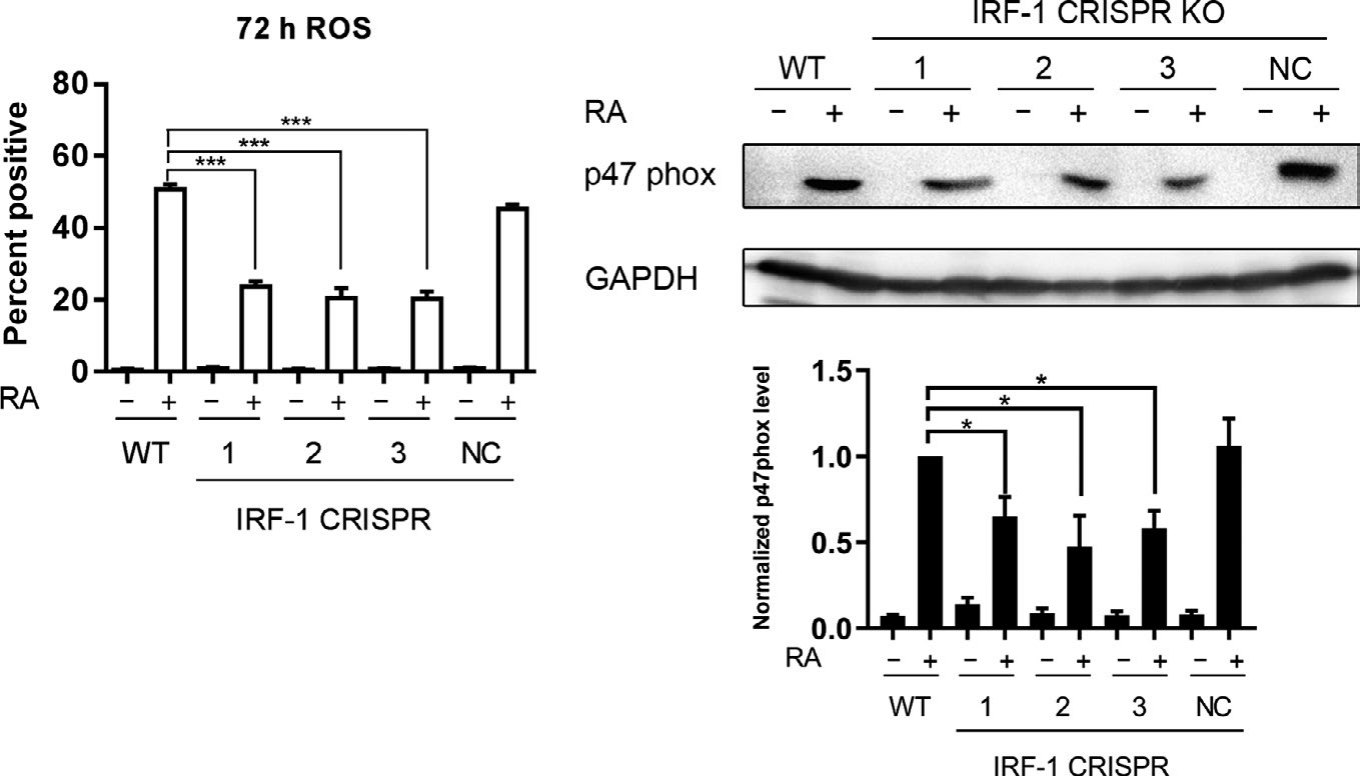
Depletion of interferon regulatory factor‐1 (IRF‐1)‐suppressed RA‐induced reactive oxygen species (ROS) through decreasing p47phox expression. Wild‐type and CRISPR‐derived HL‐60 cells were treated with 1 μmol/L RA as indicated for 72 h and analyzed for (A) the stimulated ROS production with NBT assay and (B) the protein level of p47phox with western blot. The relative level of p47phox against GAPDH was calculated with ImageJ. *indicates statistical significance with *P* < .05 for n = 3

Besides the induction of CD38, CD11b, and G1/G0 cell cycle arrest, stimulated reactive oxygen species (ROS) production is a marker for RA‐induced functionally mature HL‐60 cells. RA treatment induces p47phox expression in HL‐60 cells, which is an important component of the NADPH oxidase complex responsible for ROS production.^38^ To measure the stimulated ROS level in the IRF‐1 knockout and control cells, TPA was used to activate inducible oxidative metabolism; ie, ROS production. The resulting ROS was detected using nitroblue tetrazolium (NBT) which superoxide reduces to formazan, a blue‐black precipitate that is microscopically visible in the cells.^31^ RA treatment induced ROS in approximately 50% of the wild‐type parental cells which decreased to 20% in the three IRF‐1 knockout cells (Figure 4A). We further tested whether the p47phox expression was affected. Consistent with the observed ROS responses, RA‐induced p47phox expression was significantly decreased in IRF‐1 knockout cells compared with parental WT cells (Figure 4B). Collectively, these data suggest that the depletion of IRF‐1 diminishes RA‐induced p47phox expression with consequential attenuation of ROS production.

### 3.5 IRF‐1 disruption suppressed D3‐induced ROS without affecting differentiation

**FIGURE 5:**
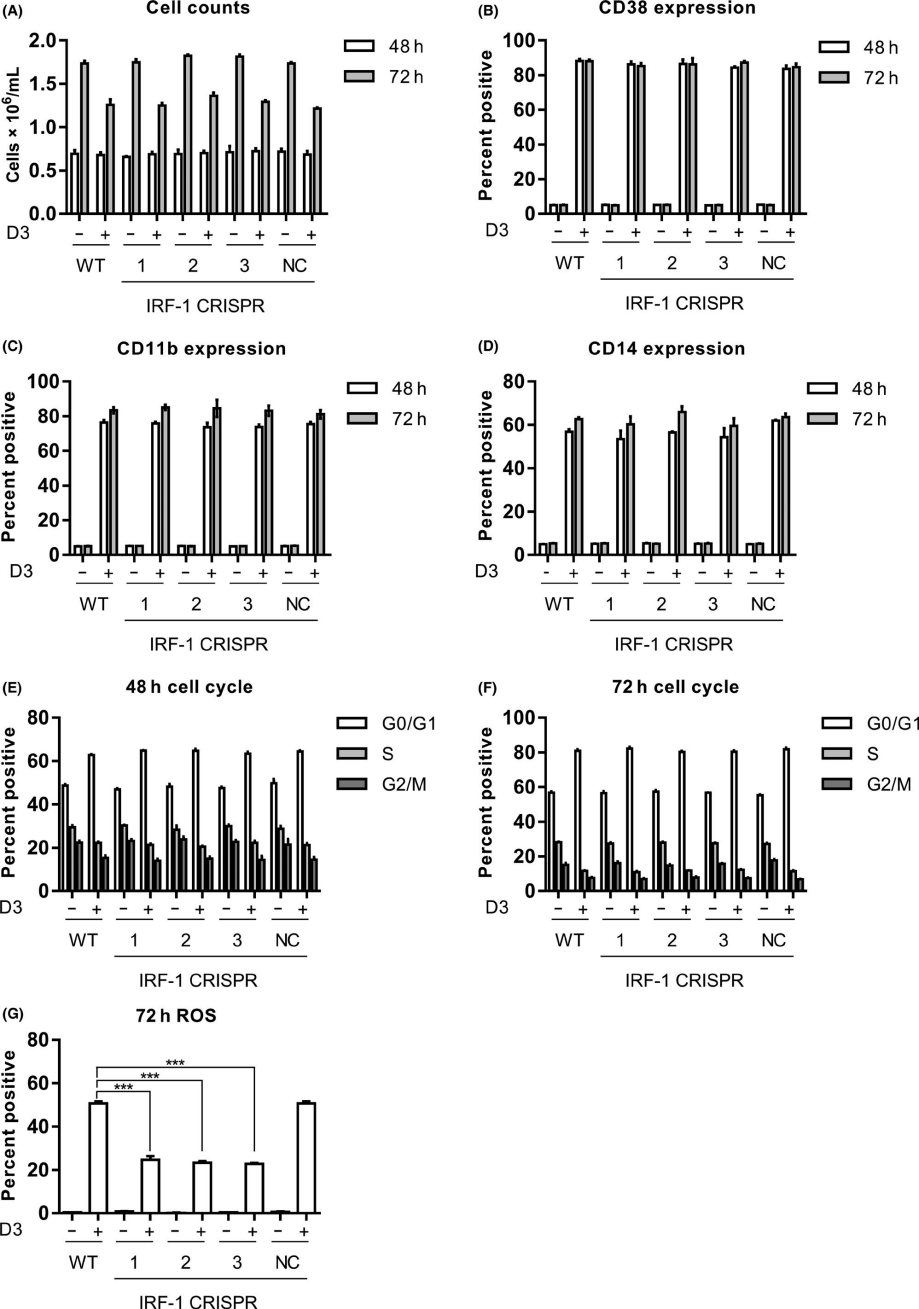
IRF‐1 disruption suppressed D3‐induced reactive oxygen species (ROS) without affecting differentiation. Wild‐type and CRISPR‐derived HL‐60 cells were treated with 0.5 uM 1,25(OH)_2_D3 as indicated for 48 and 72 h, the cells were collected for analysis of (A) cell number, (B) CD38 expression, (C) CD11b expression, (D) CD14 expression, (E) 48 h cell cycle phase distribution, (F) 72 h cell cycle phase distribution, and (G) stimulated ROS production

Since we have found that IRF‐1 was dispensable for RA‐induced differentiation of HL‐60 cells into granulocyte‐like cells, we explored whether IRF‐1 might play a more crucial role in monocyte‐like cell differentiation. To test this, IRF‐1 knockout and wild‐type cells were treated with 1,25(OH)_2_D3 to induce monocytic differentiation. Cell density, induction of CD38, CD11b, and CD14, which was a specific marker for monocyte‐like cells, as well as G1/G0 cell cycle arrest of the treated cell populations were measured after 48 and 72 hours of 1,25(OH)_2_D3 treatment.^39^ As for RA treatment, loss of IRF‐1 did not affect 1,25(OH)_2_D3‐induced differentiation or G1/G0 cell cycle arrest. Functional differentiation measured by stimulated ROS level induced by 1,25(OH)_2_D3 was also measured, and the IRF‐1 knockout cells exhibited much lower ROS compared to control cells (Figure 5). In conclusion, these data indicated that as for RA, IRF‐1 played a role in 1,25(OH)_2_D3‐stimulated ROS level but was not required for monocytic differentiation. As for RA, this was surprising since interferon is a well‐known regulator of monocytic lineage cells.

## 4 DISCUSSION

This study analyzed the dependence of RA‐induced myeloid differentiation of a non‐APL AML cell, a myeloblastic leukemia cell (HL‐60), on IRF‐1. HL‐60 is a myelo‐monocytic (granulocyte‐monocyte) precursor cell that bears fidelity to a RA‐responsive t(15,17)‐negative subtype of AML.^16^, ^40^ Interferon is a regulator of myelo‐monopoiesis. The primary mediator of the cellular effects of interferon is the IRF‐1 transcription factor. RA‐induced myeloid differentiation of HL‐60 cells involves the formation of a signalsome that includes a number of canonically cytoplasmic signaling molecules as well as the IRF‐1 transcription factor.^23^, ^28^ Signalsome signaling, causing the nuclear translocation of Raf‐1, enables transcriptional activation by RAR/RXR at target genes to drive differentiation.^32^, ^41^, ^42^ IRF‐1 is not expressed in HL‐60 cells until RA treated when expression becomes prominent.^28^ Ectopic expression of IRF‐1 results in stable transfectants where RA‐induced differentiation is enhanced.^23^ Likewise for 1,25(OH)_2_D3‐induced monocytic differentiation of these cells.^39^, ^43^ These findings point to the importance of IRF‐1 in HL‐60 cell differentiation. Indeed, it motivates the conjecture that induced IRF‐1 expression is the trigger for formation of the differentiation‐driving signalsome. We thus disrupted the IRF‐1 gene using the CRISPR‐Cas9 system in HL‐60 cells and evaluated how this affected RA and 1,25(OH)_2_D3‐induced differentiation. We analyzed cell differentiation, measuring: cell density, induction of CD38, CD11b, CD14, and G1/G0 cell cycle arrest. Surprisingly, we found that loss of IRF‐1 did not compromise expression of prominent signalsome constituents which are RA regulated and known contributors to the signaling seminal to differentiation. Nor was RA‐induced expression of the PU.1 transcription factor affected by the IRF‐1 KO. Furthermore, we found that loss of IRF‐1 did not affect RA or 1,25(OH)2D3 induced HL‐60 cell differentiation. This finding is unanticipated given the historical perception of the dependence of both RA action and myelo‐monopoiesis on interferon.

IRF‐1 loss did, however, adversely affect inducible oxidative metabolism, a functional marker for mature myelo‐monocytic cells. We found that without IRF‐1, inducible oxidative metabolism was crippled. The stimulated ROS level decreased dramatically. Considering the importance of ROS in signaling and antimicrobial activity, our data affirmed the role of IRF‐1 in one of the prominent functions of mature myelo‐monocytic cells, if not in driving differentiation. The muted ROS response probably reflected crippling of the induced expression of a component of the NADPH oxidase complex responsible for inducible oxidative metabolism, p47phox, by loss of IRF‐1.

The above is consistent with the notion that IRF‐1 supported RA‐induced p47phox upregulation in HL‐60 cells. However, it is not consistent with previous reports that PU.1, but not IRF‐1, was essential for p47phox promoter activation in HL‐60 cells since we find here that induced PU.1 expression was unaffected by IRF‐1 loss.^44^ Although it has been reported before that IRF‐1 regulated the expression of PU.1 in mice,^37^ depletion of IRF‐1 did not affect RA‐induced PU.1 level in human HL‐60 cells. However, in one report IRF‐1 formed a complex with PU.1 and IFN consensus‐binding protein (ICSBP) to mediate the expression of p67phox and gp91phox, two other important components of the NADPH oxidase complex.^45^ IRF‐1 also may cooperate with PU.1 to regulate p47phox expression in HL‐60 cells. In another study, however, it was found that when oligonucleotides competing for PU.1‐DNA binding were added to cultured monocytes, the expression of gp91phox and p22phox induced by IFN‐γ was suppressed, while p47phox expression and O2– production were not affected. This study highlighted the importance of IRF‐1 in directly regulating p47phox expression and O2– production in mature leukemia cells.^46^ Hence, there could be multiple compensatory effects in signal integration at the p47phox promoter, which can be studied in the future.

The results found are unanticipated, and there are potential caveats to the present study. Firstly, for the CRISPR‐Cas9‐mediated disruption of IRF‐1, we used a pool instead of single clone. It is possible that some IRF‐1‐positive cells still survived in the pool and affected the results, eg, KO3 (Figure 1C). However, the majority of IRF‐1 has been depleted in KO1 and KO2. And the results were consistent among these three cell lines, suggesting that the conclusion that depletion of IRF‐1 did not affect RA or 1,25(OH)_2_D3 induced differentiation is convincing. Moreover, unlike the single clone, the pooled cell population includes various sequences after the double‐strand break (DSB) repair, thereby avoiding potentially idiosyncratic results specific to a single sequence. Secondly, the IRF‐1 belongs to the IRF family, which has 10 members. And the IRF members share a highly conserved N terminal for DNA binding. It is possible that other IRF members may compensate for the loss of IRF‐1 in HL‐60 cell differentiation.^47^ For example, IRF‐2 was responsible for the transcriptional activation of caspase‐4 in noncanonical inflammasome‐mediated pyroptotic cell death. However, when IRF‐2 was missing, IRF‐1 substituted to maintain the transcription of caspase‐4.^48^ Accordingly, it is of great interest to study whether other members of IRF family also play roles in RA‐induced HL‐60 differentiation in the future.

## DISCLOSURES

None.

## AUTHOR CONTRIBUTIONS

K. ZHU, J. YUE, and A. YEN designed the experiments. K. ZHU conducted the experiments and data analysis; K. ZHU wrote the manuscript; and J. YUE and A. YEN edited and approved the manuscript.

## Supporting information

Figure S1

Figure S2

Figure S3

Supplementary Material

## References

[1] Rego EM , He LZ , Warrell RP , et al. Retinoic acid (RA) and As2O3 treatment in transgenic models of acute promyelocytic leukemia (APL) unravel the distinct nature of the leukemogenic process induced by the PML‐RARα and PLZF‐RARα oncoproteins. Proc Natl Acad Sci. 2000;97(18):10173‐10178. 10954752 10.1073/pnas.180290497 PMC27786

[2] Dalton WTJ , Ahearn MJ , McCredie KB , et al. HL‐60 cell line was derived from a patient with FAB‐M2 and not FAB‐M3. Blood. 1988;71(1):242‐247. 3422031

[3] Bunaciu RP , Yen A . Retinoid chemoprevention: who can benefit? Current pharmacology reports, Vol. 1. 2015:391‐400. 10.1007/s40495-015-0036-8 PMC4628910 26539342

[4] Jensen HA , Bunaciu RP , Ibabao CN , et al. Retinoic acid therapy resistance progresses from unilineage to bilineage in HL‐60 leukemic blasts. PLoS ONE. 2014;9(6):e98929. 24922062 10.1371/journal.pone.0098929 PMC4055670

[5] McCarthy DM , San Miguel JF , Freake HC , et al. 1, 25‐dihydroxyvitamin D3 inhibits proliferation of human promyelocytic leukaemia (HL60) cells and induces monocyte‐macrophage differentiation in HL60 and normal human bone marrow cells. Leuk Res. 1983;7(1):51‐55. 6682163 10.1016/0145-2126(83)90057-7

[6] Vieth R . The mechanisms of vitamin D toxicity. Bone and mineral. 1990;11(3):267‐272. 2085680 10.1016/0169-6009(90)90023-9

[7] Tallman MS , Andersen JW , Schiffer CA , et al. All‐trans‐retinoic acid in acute promyelocytic leukemia. N Engl J Med. 1997;337(15):1021‐1028. 9321529 10.1056/NEJM199710093371501

[8] Frankel SR , Eardley A , Lauwers G , Weiss M , Warrell RP . The retinoic acid syndrome in acute promyelocytic leukemia. Ann Intern Med. 1992;117(4):292‐296. 1637024 10.7326/0003-4819-117-4-292

[9] Chelbi‐Alix M , Pelicano L . Retinoic acid and interferon signaling cross talk in normal and RA‐resistant APL cells. Leukemia. 1999;13(8):1167. 10450744 10.1038/sj.leu.2401469

[10] Clarke N , Jimenez‐Lara AM , Voltz E , et al. Tumor suppressor IRF‐1 mediates retinoid and interferon anticancer signaling to death ligand TRAIL. EMBO J. 2004;23(15):3051‐3060. 15241475 10.1038/sj.emboj.7600302 PMC514919

[11] Miyamoto M , Fujita T , Kimura Y , et al. Regulated expression of a gene encoding a nuclear factor, IRF‐1, that specifically binds to IFN‐β gene regulatory elements. Cell. 1988;54(6):903‐913. 3409321 10.1016/s0092-8674(88)91307-4

[12] Schaper F , Kirchhoff S , Posern G , et al. Functional domains of interferon regulatory factor I (IRF‐1). Biochem J. 1998;335(Pt 1):147. 9742224 10.1042/bj3350147 PMC1219763

[13] Schwartz J , Shajahan A , Clarke R . The role of interferon regulatory factor‐1 (IRF1) in overcoming antiestrogen resistance in the treatment of breast cancer. Int J Breast Cancer. 2011;2011:1–9. 10.4061/2011/912102 PMC3262563 22295238

[14] Willman C , Sever C , Pallavicini M , et al. Deletion of IRF‐1, mapping to chromosome 5q31. 1, in human leukemia and preleukemic myelodysplasia. Science. 1993;259(5097):968‐971. 8438156 10.1126/science.8438156

[15] Matsuyama T , Kimura T , Kitagawa M , et al. Targeted disruption of IRF‐1 or IRF‐2 results in abnormal type I IFN gene induction and aberrant lymphocyte development. Cell. 1993;75(1):83‐97. 8402903

[16] Bunaciu RP , MacDonald RJ , Gao F , et al. Potential for subsets of wt‐NPM1 primary AML blasts to respond to retinoic acid treatment. Oncotarget. 2018;9(3):4134. 29423110 10.18632/oncotarget.23642 PMC5790527

[17] Yen A , Forbes ME . C‐myc down regulation and precommitment in HL‐60 cells due to bromodeoxyuridine. Can Res. 1990;50(5):1411‐1420. 2302706

[18] Nasr R , Guillemin M‐C , Ferhi O , et al. Eradication of acute promyelocytic leukemia‐initiating cells through PML‐RARA degradation. Nat Med. 2008;14(12):1333. 19029980 10.1038/nm.1891

[19] Tasseff R , Jensen HA , Congleton J , et al. An effective model of the retinoic acid induced HL‐60 differentiation program. Sci Rep. 2017;7(1):14327. 29085021 10.1038/s41598-017-14523-5 PMC5662654

[20] Yen A , Roberson MS , Varvayanis S , Lee AT . Retinoic acid induced mitogen‐activated protein (MAP)/extracellular signal‐regulated kinase (ERK) kinase‐dependent MAP kinase activation needed to elicit HL‐60 cell differentiation and growth arrest. Can Res. 1998;58(14):3163‐3172. 9679985

[21] Hong H‐Y , Varvayanis S , Yen A . Retinoic acid causes MEK‐dependent RAF phosphorylation through RARα plus RXR activation in HL‐60 cells. Differentiation. 2001;68(1):55‐66. 11683493 10.1046/j.1432-0436.2001.068001055.x

[22] Congleton J , Shen M , MacDonald R , et al. Phosphorylation of c‐Cbl and p85 PI3K driven by all‐trans retinoic acid and CD38 depends on Lyn kinase activity. Cell Signal. 2014;26(7):1589‐1597. 24686085 10.1016/j.cellsig.2014.03.021 PMC4039659

[23] Shen M , Bunaciu RP , Congleton J , et al. Interferon regulatory factor‐1 binds c‐Cbl, enhances mitogen activated protein kinase signaling and promotes retinoic acid‐induced differentiation of HL‐60 human myelo‐monoblastic leukemia cells. Leukemia & lymphoma. 2011;52(12):2372‐2379. 21740303 10.3109/10428194.2011.603449 PMC3989140

[24] Shen M , Yen A . c‐cbl interacts with cd38 and promotes retinoic acid–induced differentiation and g0 arrest of human myeloblastic leukemia cells. Can Res. 2008;68(21):8761‐8769. 10.1158/0008-5472.CAN-08-1058 PMC4896297 18974118

[25] Shen M , Yen A . c‐Cbl tyrosine kinase‐binding domain mutant G306E abolishes the interaction of c‐Cbl with CD38 and fails to promote retinoic acid‐induced cell differentiation and G0 arrest. J Biol Chem. 2009;284(38):25664‐25677. 19635790 10.1074/jbc.M109.014241 PMC2757968

[26] Yen A , Varvayanis S , Smith JL , et al. Retinoic acid induces expression of SLP‐76: expression with c‐FMS enhances ERK activation and retinoic acid‐induced differentiation/G0 arrest of HL‐60 cells. Eur J Cell Biol. 2006;85(2):117‐132. 16439309 10.1016/j.ejcb.2005.09.020

[27] Congleton J , Jiang H , Malavasi F , et al. ATRA‐induced HL‐60 myeloid leukemia cell differentiation depends on the CD38 cytosolic tail needed for membrane localization, but CD38 enzymatic activity is unnecessary. Exp Cell Res. 2011;317(7):910‐919. 21156171 10.1016/j.yexcr.2010.12.003 PMC3601376

[28] Matikainen S , Ronni T , Hurme M , et al. Retinoic acid activates interferon regulatory factor‐1 gene expression in myeloid cells. Blood. 1996;88(1):114‐123. 8704165

[29] Green WB , Slovak ML , Chen I‐M , et al. Lack of IRF‐1 expression in acute promyelocytic leukemia and in a subset of acute myeloid leukemias with del (5)(q31). Leukemia. 1999;13(12):1960. 10602416 10.1038/sj.leu.2401596

[30] MacDonald RJ , Shrimp JH , Jiang H , et al. Probing the requirement for CD38 in retinoic acid‐induced HL‐60 cell differentiation with a small molecule dimerizer and genetic knockout. Sci Rep. 2017;7(1):17406. 29234114 10.1038/s41598-017-17720-4 PMC5727258

[31] Yen A , Albright KL . Evidence for cell cycle phase‐specific initiation of a program of HL‐60 cell myeloid differentiation mediated by inducer uptake. Can Res. 1984;44(6):2511‐2515. 6327017

[32] Geil WM , Yen A . Nuclear Raf‐1 kinase regulates the CXCR 5 promoter by associating with NFAT c3 to drive retinoic acid‐induced leukemic cell differentiation. FEBS J. 2014;281(4):1170‐1180. 24330068 10.1111/febs.12693 PMC4057990

[33] Smith J , Bunaciu RP , Reiterer G , et al. Retinoic acid induces nuclear accumulation of Raf1 during differentiation of HL‐60 cells. Exp Cell Res. 2009;315(13):2241‐2248. 19298812 10.1016/j.yexcr.2009.03.004 PMC2696568

[34] Liu Y‐C , Elly C , Yoshida H , et al. Activation‐modulated association of 14–3–3 proteins with Cbl in T cells. J Biol Chem. 1996;271(24):14591‐14595. 8663231 10.1074/jbc.271.24.14591

[35] Congleton J , MacDonald R , Yen A . Src inhibitors, PP2 and dasatinib, increase retinoic acid‐induced association of Lyn and c‐Raf (S259) and enhance MAPK‐dependent differentiation of myeloid leukemia cells. Leukemia. 2012;26(6):1180. 22182854 10.1038/leu.2011.390 PMC3310950

[36] Katagiri K , Yokoyama KK , Yamamoto T , et al. Lyn and Fgr protein‐tyrosine kinases prevent apoptosis during retinoic acid‐induced granulocytic differentiation of HL‐60 cells. J Biol Chem. 1996;271(19):11557‐11562. 8626717 10.1074/jbc.271.19.11557

[37] Testa U , Stellacci E , Pelosi E , et al. Impaired myelopoiesis in mice devoid of interferon regulatory factor 1. Leukemia. 2004;18(11):1864. 15385939 10.1038/sj.leu.2403472

[38] MacDonald RJ , Bunaciu RP , Ip V , et al. Src family kinase inhibitor bosutinib enhances retinoic acid‐induced differentiation of HL‐60 leukemia cells. Leukemia & lymphoma. 2018;59(12):2941‐2951. 29569971 10.1080/10428194.2018.1452213 PMC6151292

[39] Supnick HT , Bunaciu RP , Yen A . The c‐Raf modulator RRD‐251 enhances nuclear c‐Raf/GSK‐3/VDR axis signaling and augments 1, 25‐dihydroxyvitamin D3‐induced differentiation of HL‐60 myeloblastic leukemia cells. Oncotarget. 2018;9(11):9808. 29515772 10.18632/oncotarget.24275 PMC5839403

[40] Bunaciu RP , MacDonald RJ , Jensen HA , et al. Retinoic acid and 6‐formylindolo (3, 2‐b) carbazole (FICZ) combination therapy reveals putative targets for enhancing response in non‐APL AML. Leukemia & lymphoma. 2019;60(7):1697‐1708. 30570341 10.1080/10428194.2018.1543880 PMC6586535

[41] Bunaciu RP , Yen A . Activation of the Aryl hydrocarbon receptor AhR promotes retinoic acid‐induced differentiation of myeloblastic leukemia cells by restricting expression of the stem cell transcription factor Oct4. Can Res. 2011;71(6):2371‐2380. 10.1158/0008-5472.CAN-10-2299 PMC3391168 21262915

[42] Wang J , Yen A . A novel retinoic acid‐responsive element regulates retinoic acid‐induced BLR1 expression. Mol Cell Biol. 2004;24(6):2423‐2443. 14993281 10.1128/MCB.24.6.2423-2443.2004 PMC355834

[43] Shen M , Yen A . Nicotinamide cooperates with retinoic acid and 1, 25‐dihydroxyvitamin D3 to regulate cell differentiation and cell cycle arrest of human myeloblastic leukemia cells. Oncology. 2009;76(2):91‐100. 19127080 10.1159/000188664 PMC2826433

[44] Li SL , Valente AJ , Zhao SJ , Clark RA PU 1. is essential for p47 phox promoter activity in myeloid cells. J Biol Chem. 1997;272(28):17802‐17809. 9211934 10.1074/jbc.272.28.17802

[45] Eklund EA , Kakar R . Recruitment of CREB‐binding protein by PU. 1, IFN‐regulatory factor‐1, and the IFN consensus sequence‐binding protein is necessary for IFN‐γ‐induced p67phox and gp91phox expression. J Immunol. 1999;163(11):6095‐6105. 10570299

[46] Dusi S , Donini M , Lissandrini D , et al. Mechanisms of expression of NADPH oxidase components in human cultured monocytes: role of cytokines and transcriptional regulators involved. Eur J Immunol. 2001;31(3):929‐938. 11241298 10.1002/1521-4141(200103)31:3<929::aid-immu929>3.0.co;2-m

[47] Alsamman K , El‐Masry OS . Interferon regulatory factor 1 inactivation in human cancer. Biosci Rep. 2018;38(3). 10.1042/BSR20171672 PMC5938431 29599126

[48] Benaoudia S , Martin A , Puig Gamez M , et al. A genome‐wide screen identifies IRF2 as a key regulator of caspase‐4 in human cells. EMBO Rep. 2019;20(9):e48235. 31353801 10.15252/embr.201948235 PMC6727027

