# Supplementary figures and images for: Depleting interferon regulatory factor‐1(IRF‐1) with CRISPR/Cas9 attenuates inducible oxidative metabolism without affecting RA‐induced differentiation in HL‐60 human AML cells
Source: FASEB Bioadv. 2020 May 22;2(6):354–64. doi: 10.1096/fba.2020-00004 (PMC7325585; doi:10.1096/fba.2020-00004)

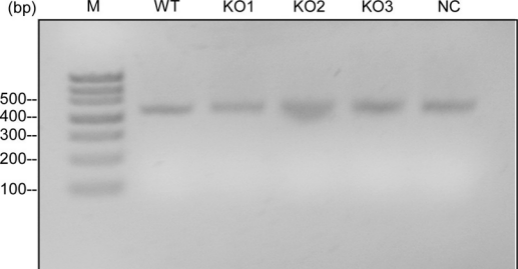

Figure S1

Supplement: Supplementary file 1 — Figure S1 [file FBA2-2-354-s001.pdf]

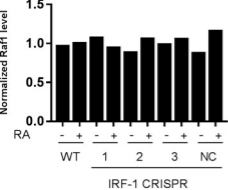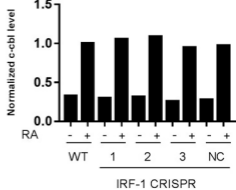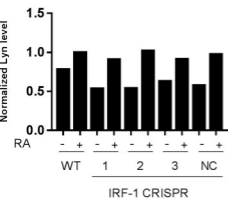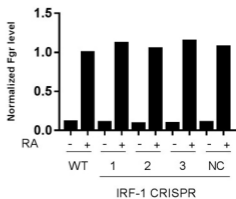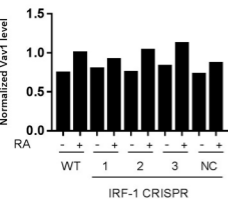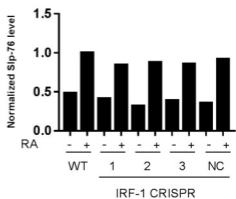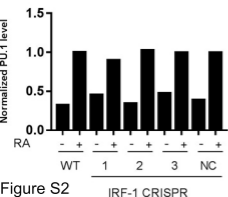

Figure S2

Supplement: Supplementary file 2 — Figure S2 [file FBA2-2-354-s002.pdf]

A

## CD38 expression

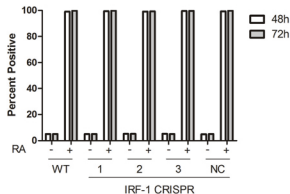

B

## CD11b expression

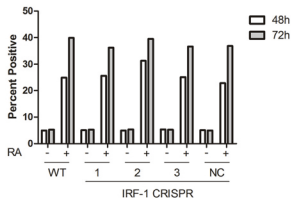

C

## 48h Cell Cycle

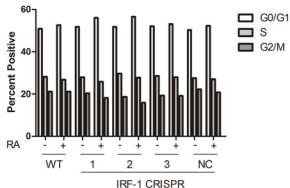

D

## 72h Cell Cycle

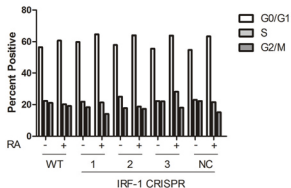

Figure S3

Supplement: Supplementary file 3 — Figure S3 [file FBA2-2-354-s003.pdf]
