# Supplementary material for: Depleting interferon regulatory factor‐1(IRF‐1) with CRISPR/Cas9 attenuates inducible oxidative metabolism without affecting RA‐induced differentiation in HL‐60 human AML cells
Source: FASEB Bioadv. 2020 May 22;2(6):354–64. doi: 10.1096/fba.2020-00004 (PMC7325585; doi:10.1096/fba.2020-00004)
Supplement: Supplementary file 4 — Supplementary Material [file FBA2-2-354-s004.pdf]

Figure legend

Figure S1. The genomic DNA purified from Wild-type and CRISPR-derived HL60 cells before genomic cleavage assay.

Figure S2. The relative levels of Raf1, c-cbl, Lyn, Fgr, Vav1, Slp-76 and PU.1 in Figure 2 were calculated with ImageJ.

Figure S3. Wild-type and CRISPR-derived HL-60 cells were treated with 0.1μM RA as indicated for 48 h and 72h, and the cells were collected for analysis of **(A)** CD38 and **(B)** CD11b expression, **(C)** 48h and **(D)** 72h cell cycle phase distribution.
